# Characterization of Desmoglein Expression in the Normal Prostatic Gland. Desmoglein 2 Is an Independent Prognostic Factor for Aggressive Prostate Cancer

**DOI:** 10.1371/journal.pone.0098786

**Published:** 2014-06-04

**Authors:** Alison G. Barber, Mireia Castillo-Martin, Dennis M. Bonal, Benjamin A. Rybicki, Angela M. Christiano, Carlos Cordon-Cardo

**Affiliations:** 1 Department of Genetics and Development, Columbia University, New York, New York, United States of America; 2 Department of Dermatology, Columbia University, New York, New York, United States of America; 3 Department of Pathology and Cell Biology, Columbia University, New York, New York, United States of America; 4 Department of Urology, Columbia University, New York, New York, United States of America; 5 Herbert Irving Comprehensive Cancer Center, Columbia University, New York, New York, United States of America; 6 Department of Public Health Sciences, Henry Ford Health System, Detroit, Michigan, United States of America; 7 Department of Pathology, Icahn School of Medicine at Mount Sinai, New York, New York, United States of America; Northern Institute for Cancer Research, United Kingdom

## Abstract

**Purpose:**

The expression of desmogleins (DSGs), which are known to be crucial for establishing and maintaining the cell-cell adhesion required for tissue integrity, has been well characterized in the epidermis and hair follicle; however, their expression in other epithelial tissues such as prostate is poorly understood. Although downregulation of classical cadherins, such as E-cadherin, has been described in prostate cancer tissue samples, the expression of desmogleins has only been previously reported in prostate cancer cell lines. In this study we characterized desmoglein expression in normal prostate tissues, and further investigated whether Desmoglein 2 (DSG2) expression specifically can serve as a potential clinical prognostic factor for patients diagnosed with primary prostate cancer.

**Experimental Design:**

We utilized immunofluorescence to examine DSG2 expression in normal prostate (*n* = 50) and in a clinically well-characterized cohort of prostate cancer patients (*n* = 414). Correlation of DSG2 expression with clinico-pathological characteristics and biochemical recurrence was analyzed to assess its clinical significance.

**Results:**

These studies revealed that DSG2 and DSG4 were specifically expressed in prostatic luminal cells, whereas basal cells lack their expression. In contrast, DSG1 and DSG3 were not expressed in normal prostate epithelium. Further analyses of DSG2 expression in prostate cancer revealed that reduced levels of this biomarker were a significant independent marker of poor clinical outcome.

**Conclusion:**

Here we report for the first time that a low DSG2 expression phenotype is a useful prognostic biomarker of tumor aggressiveness and may serve as an aid in identifying patients with clinically significant prostate cancer.

## Introduction

Desmosomes are a type of cell-cell anchoring junction found in tissues that undergo a high degree of mechanical stress, and loss of desmosomal adhesion is associated with diseases affecting the skin, hair, and heart [1]–[13]. In the desmosome, the cadherin family of proteins is represented by the so-called “non-classical” cadherins, which include desmogleins (DSG 1–4) and desmocollins (DSC 1–3) [14]. Desmogleins and desmocollins play a dual role in the formation of desmosomes. In the extracellular space, desmogleins and desmocollins on opposing cells interact in a heterophilic calcium-dependent manner via their cadherin repeat domains. Intracellularly, the cadherins bind to plakoglobin and plakophilin, which in turn bind to desmoplakin. Desmoplakin then binds to intermediate filaments, thereby securing the entire structure to the cytoskeleton [15], [16]. While clearly important in maintaining the integrity of adult tissues, the role of desmosomal cadherins in cancer progression is less well understood. Loss of heterozygosity near the chromosomal region containing the desmosomal cadherin gene cluster has been reported in esophageal cancer as well as head and neck squamous cell carcinoma [17], [18]. Alterations in the expression of desmogleins have been reported for a variety of cancers. Reduced expression of DSG2 has been reported in gastric and pancreatic carcinomas [19]–[21]. Conversely, overexpression of DSG2 and DSG3 has been reported in squamous cell carcinomas of the skin as well as head and neck cancer, respectively [22], [23].

The expression of desmosomal cadherins has been thoroughly characterized in the epidermis, hair follicle, and arachnoidal tissue. While the expression of DSG2 is known to be ubiquitous in all desmosome-forming tissues, the expression of the remaining desmosomal cadherins outside of the epidermis and hair follicle is poorly understood [24]. A study by Whittock and Bower in 2003 utilized RT-PCR to analyze the expression of *DSG4* in a panel of tissues and found that *DSG4* could be detected in several tissues including the prostate, suggesting that the desmoglein expression profile of the prostate may extend beyond the ubiquitous expression of DSG2 [25]. The downregulation or loss of cell-cell adhesion proteins — such as the classical cadherin, E-cadherin — is a common feature of a variety of cancers, including prostate cancer, and can be caused by a variety of different mechanisms [26], [27]. Conversely, though the aberrant expression of desmogleins has been reported in several types of cancer, the expression of these cadherins in prostate cancer has only been reported in cell lines to date [28]–[30]. In this study we characterize for the first time the expression of desmogleins in normal human prostate tissue specimens and determine the specific cell type in which prostate specific desmogleins are expressed. We then analyze the expression of DSG2 in a well-characterized prostate cancer patient cohort, and examine the association between DSG2 expression and patients' clinical outcome. Our results reveal that DSG2 and DSG4 are specifically expressed in the luminal cells of normal human prostate, whereas DSG1 and DSG3 are not expressed in the prostate epithelium. Further, reduced expression of DSG2 was found to be independently associated with a shorter biochemical recurrence, highlighting the potential utility of DSG2 expression as a prognostic biomarker of prostate cancer aggressiveness.

## Materials and Methods

### Ethics statement

Frozen normal human prostate tissue slides corresponding to prostatic transitional zone with histologically confirmed areas of normal glands from patients who underwent a radical prostatectomy were obtained anonymously from the Columbia Tumor Bank Service in accordance with the Institutional Review Board of Columbia University Protocol #AAAB2447. The TMAs utilized in this study were built in the Cordon-Cardo laboratory, and were generated from 414 radical prostatectomy cases, originally collected between September 2000 and January 2005 at the Henry Ford Health System in Detroit, following an Institutional Review Board's approved protocol #1018 [31]. Each participant completed a brief telephone interview (demographics and health history), a food frequency questionnaire, and a face-to-face interview on job related exposures. In addition, all participants provided a blood sample for genetic analyses and for PSA testing. Enrollment closed in spring of 2005, and the IRB remains open for PSA follow-up (non-patient contact) for study subjects.

### Cell Culture and RNA Isolation

The benign prostate epithelial BPH-1 cell line (a generous gift from Dr. Ralph Buttyan, and commercially available through the German Collection of Microorganisms and Cell Cultures; DSMZ, Braunschweig, Germany) was cultured in RPMI with 10% fetal bovine serum (FBS) (Invitrogen, Carlsbad, CA). Three human metastatic prostate cancer cell lines were used in this study: DU145, PC3 and LNCaP. The DU145 cell line (ATCC, Manassas, VA) was cultured in MEM with 10% FBS (Invitrogen, Carlsbad, CA). The PC3 cell line (ATCC, Manassas, VA) was cultured in F12K with 10% FBS (Invitrogen, Carlsbad, CA). The LNCaP cell line (ATCC, Manassas, VA) was cultured in RPMI with 10% FBS (Invitrogen, Carlsbad, CA). To isolate RNA, cells were washed in 1X PBS, trypsinized, and pelleted via centrifugation. Cells were grown four days past confluency prior to RNA isolation as it has been previously reported that their full desmoglein expression profile is reached at this time point [32]. The cell pellets were resuspended in 1X PBS and then pelleted via centrifugation to wash. This wash step was repeated twice to remove all traces of media prior to harvesting RNA. RNA was then harvested using the RNeasy Mini Kit and QIAshredder following the manufacturer's protocol entitled “Purification of Total RNA from Animal Cells Using Spin Technology” (Qiagen, Valencia, CA).

### Antibodies

Anti-DSG3 (clone 5H10) and DSG1 (clone 27B2) mouse monoclonal antibodies have been previously described and were given to us as a generous gift from Dr. James Wahl; both were used at a dilution of 1∶10 [33]. Anti-DSG2 mouse monoclonal antibody (clone 10G11) was purchased from ARP, Inc (Belmont, MA) and used at a dilution of 1∶50 for immunofluorescence analysis of normal prostate tissue and cell lines; anti-DSG1/2 mouse monoclonal antibody (clone DG3.10) was purchased from Fitzgerald (Acton, MA) and used at a dilution of 1∶20 for TMAs. Anti-DSG4 guinea pig polyclonal antibody was generated by Dr. Lutz Langbein and given to us as a generous gift, this antibody was used at dilution of 1∶2000. Anti-cytokeratin 14 (CK14) rabbit polyclonal antibody was purchased from Covance (Princeton, NJ) and used at a dilution of 1∶1000. Anti-PSA mouse monoclonal antibody was purchased from Dako (Carpinteria, CA) and used at a dilution of 1∶20. Anti-PSA rabbit monoclonal antibody was purchased from Abcam (Cambridge, MA) and used at a dilution of 1∶200. Anti-cytokeratins 8/18 (CK8/18) guinea pig polyclonal antibody was purchased from Progen (Heidelberg, Germany) and used at a dilution 1∶100. Alexa Fluor 594 or Alexa Fluor 488 secondary antibodies were purchased from Invitrogen (Carlsbad, CA) and were used at a dilution of 1∶600.

### RT-PCR

Total RNA from human normal skin and normal prostate (both sourced from a single donor) was purchased commercially (Agilent Technologies Inc., Santa Clara, CA). First strand cDNA was made using Oligo dT and the SuperScript II First-Strand Synthesis System (Invitrogen, Carlsbad, CA) following the manufacturer's instructions. PCR was performed using Platinum PCR Supermix (Invitrogen, Carlsbad, CA) and primers for the transcripts of interest (summarized in Table S1). The following PCR protocol was used: step 1: 94°C for 3 min; step 2: 94°C for 30 sec, 56°C for 45 sec, 72°C for 2 min; step 3: 72°C for 10 min; repeat step 2 for 34 cycles. PCR products were electrophoresed on a 1% agarose/1X TBE gel containing ethidium bromide and visualized using Kodak Electrophoresis Documentation and Analysis System 120 Camera (Kodak, Rochester, NY).

### qRT-PCR

RNA was harvested from cell lines of interest as described. First strand cDNA was made using Oligo dT and the SuperScript III First-Strand Synthesis System (Invitrogen, Carlsbad, CA) following the manufacturer's instructions. qRT-PCR was performed on a Stratagene Mx3005P machine and analyzed using Stratagene MxPro QPCR software (Stratagene, Santa Clara, CA). All reactions were performed using QuantiTect SYBR Green PCR Master Mix (Qiagen, Valencia, CA), 200 nM primers (summarized in Table S1), and 10 ng cDNA in a 20 µL reaction volume. The following PCR reaction was used: step 1: 95°C for 10 min; step 2: 95°C for 15 sec, 60°C for 1 min; repeat step 2 for 40 cycles. All samples were run in quadruplicate, and samples were normalized against an endogenous internal control, β-actin.

### Immunofluorescence—Frozen Tissues and Cell Lines

Frozen normal human prostate tissue slides corresponding to prostatic transitional zone with histologically confirmed areas of normal glands from patients were used to characterize the antibodies to detect desmoglein expression. Cell lines were grown on glass coverslips (Fisher, Pittsburgh, PA) in 6-well tissue culture plates (BD Falcon, Bedford, MA). Slides/coverslips were fixed in cold 100% methanol for 15 min at −20°C followed by cold 100% acetone fixation for 2 min at −20°C. Slides/coverslips were washed in 1X phosphate buffered saline (PBS) with agitation, permeabilized with 1% Triton-X100 in 1X PBS at room temperature for 5 min, and washed again in 1X PBS with agitation. Slides/coverslips were incubated in 0.1% Triton X-100/1% bovine serum albumin (BSA)/1X PBS block at room temperature for 1 hour, followed by primary antibody incubation overnight at 4°C. The following day slides/coverslips were washed in 1XPBS with agitation, then a 1∶600 dilution of secondary antibody, either Alexa Fluor 594 or Alexa Fluor 488 (Invitrogen, Carlsbad, CA), was added and slides/coverslips were incubated at room temperature for 45 min. Slides/coverslips were then washed in 1XPBS with agitation, immersed briefly in water and mounted using VECTASHIELD mounting medium with DAPI (Vector Laboratories, Burlingame, CA).

### Immunofluorescence Analysis of Tissue Microarray (TMA) Sections

TMAs were built as follows: Hematoxylin & Eosin stained sections from formalin-fixed paraffin-embedded (FFPE) prostatectomy specimens were reviewed to identify viable, morphologically representative areas of normal prostate glands and acinar adenocarcinoma from which needle core samples could be taken. From each specimen, triplicate adjacent tissue cores with diameters of 0.6 mm were punched and arrayed onto a recipient paraffin block using a precision instrument (Beecher Instruments, MD). All punches were obtained from a focus with the highest Gleason Score, and the majority of punches were taken from the dominant tumor nodule — defined as the largest nodule with the highest Gleason Score and stage in a specific prostatectomy specimen. Follow-up data for all the cases regarding evidence of biological recurrence was available for this study. Consecutive five-micrometer sections of these TMA blocks were used for immunofluorescence analysis, and the first section was stained with Hematoxylin & Eosin to serve as a template for morphology. The slides were deparaffinized and rehydrated, and antigen retrieval was performed by heating slides in a steamer in citrate buffer, pH 6.0 for 15 minutes. Slides were then washed once in 1X PBS. Slides were incubated in 0.1% Triton X-100/1% BSA/1X PBS blocking serum at room temperature for 1 hour followed by primary antibody incubation overnight at 4°C. The following day, slides were washed in 1XPBS with agitation, then a 1∶600 dilution of secondary antibody, either Alexa Fluor 594 or Alexa Fluor 488 (Invitrogen, Carlsbad, CA), was added and slides were incubated at room temperature for 45 min. Slides were then washed three times in 1X PBS+0.1% Triton X with agitation, washed three times in 1X PBS, immersed briefly in water and mounted using VECTASHIELD mounting medium with DAPI (Vector Laboratories, Burlingame, CA). CK8/18 expression was used to identify all epithelial areas (both tumor and normal glands) in the cores and the expression of the proteins of interest was scored by determining the percentage of tumor cells with immunoreactivity per tissue core (from 0% to 100%), without considering intensity of signal. The evaluation was performed by a uropathologist (MCM) following previously used methodology: tumor areas in the core were identified first by DAPI, confirmed by the presence of CK8/18 and then scored by establishing a percentage of tumors cells with membrane expression of DSG2 [34].The average values of the representative cores from each patient sample were then used for statistical analyses. A positive cut-off of 60% was used to perform statistical analyses of correlation with clinico-pathological features and survival as this was the approximate median expression value observed for DSG2 in this prostate cancer cohort, as previously reported in the literature [35].

### Prostate Cancer Cohort and Clinico-pathological Characteristics

In this study we analyzed a cohort of 414 patients diagnosed with primary prostate cancer. Clinico-pathological features of these patients are summarized in Table 1. Mean age at diagnosis was 61 years (range: 41–74.7 years). Most of the patients were white (56%) or African-American (43%). Mean follow-up was 56.8 months (range: 1.4–123.6 months). Only 98 (24%) of the patients showed a biochemical relapse, defined as having two consecutive detectable rising PSA levels (>0.2 ng/ml) four weeks or more after surgery. Biochemical recurrence free survival curves corresponding to well known clinico-pathological risk factors such as Pre-surgical PSA, Pathological Stage, Gleason Score, Angiolymphatic Invasion and Perineural Invasion, are displayed in Figure S1.

**Table 1:** Clinico-pathological characteristics of patients (*n* = 414).

**Age at Diagnosis**	
Mean	61
Range	41–74.7
Unavailable	5 (1%)
**Race**	
White	230 (56%)
African American	179 (43%)
Asian/Polynesian	1 (<1%)
Hispanic	2 (<1%)
Unknown	2 (<1%)
**PSA at Diagnosis (ng/mL)**	
Mean	7
Range	0.4–51.4
<4	72 (17%)
4–10	272 (66%)
>10	66 (16%)
Unavailable	4 (<1%)
**Gleason Score**	
≤6	148 (36%)
7	177 (43%)
8	52 (13%)
≥9	25 (5%)
Unavailable	12 (3%)
**TNM Stage**	
T1, T2	329 (79%)
T3	73 (18%)
T4	10 (2%)
Unavailable	2 (<1%)
**Follow-up (months)**	
Mean	56.8
Range	1.4–123.6
**Biochemical Relapse**	
Negative	316 (76%)
Positive	98 (24%)

### Statistical Analysis

Statistical analysis was conducted using SPSS v18.0 (IBM, Armonk, NY). The Student's t-test was used to compare the expression of the protein of interest in primary prostate cancer and normal prostate tissue. Spearman's rank correlation was used to analyze the correlation between proteins of interest and clinico-pathological features. Biochemical recurrence free survival was analyzed using Kaplan-Meier survival curves, and curves were compared using the log-rank test. Multivariate analysis including DSG2 expression and clinico-pathological parameters was performed using Cox proportional hazards model. A *P*-value ≤0.05 was considered statistically significant.

## Results

### Desmogleins are Specifically Expressed in Luminal Cells of the Human Normal Prostate

**Figure 1:**
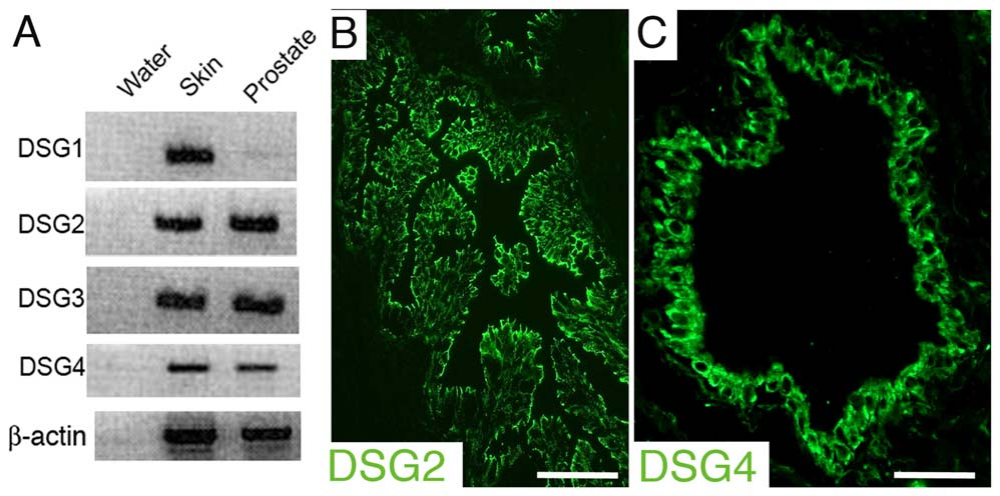
Desmoglein expression in normal human prostate. (A) Clear expression of mRNA can be detected for most desmogleins, with the exception of *DSG1* which shows only faint expression. (**B–C**) Representative immunofluorescence analyses of DSG2 (B) and DSG4 (C) at the cell border of normal human prostatic glandular epithelium. Original magnification: 200X. Scale bars correspond to 100 µm.

As the expression of desmogleins in the prostate gland had not been characterized to date, we began this study by examining the full desmoglein expression profile in normal human prostate tissue, using RT-PCR and immunofluorescence analyses. RT-PCR analysis showed that most desmogleins could be readily detected in normal human prostate at the RNA level, with the exception of *DSG1* which showed only faint mRNA expression (Figure 1A). Immunofluorescence analysis revealed that only the expression of DSG2 and DSG4 could be readily and consistently detected in the prostate epithelium at the protein level (Figure 1B–C), whereas DSG1 and DSG3 were not demonstrable in the normal prostate epithelium (Figure S2).

Having established the expression of desmogleins in normal human prostate, we aimed to determine the cell type specific expression of DSG2 and DSG4. The glandular tissue of the prostate is comprised of ducts and acini that are lined by a simple cuboidal epithelium facing the lumen of the glands. In addition to this secretory component, two other specialized cells are an integral part of the prostate glandular structures, namely the basal cells and the neuroendocrine cells [36], [37]. Luminal cells secrete, among other molecules, prostate specific antigen (PSA) and represent the main functional unit of the organ. These cells form a continuous layer that lies on top of the basal cells. The basal cells are characterized by the expression of high molecular weight cytokeratins (CKs) — such as CK14 — and p63. Dispersed throughout the acini is the third cell population, the neuroendocrine cells, the origin and function of which in the normal prostate are not yet well defined.

**Figure 2:**
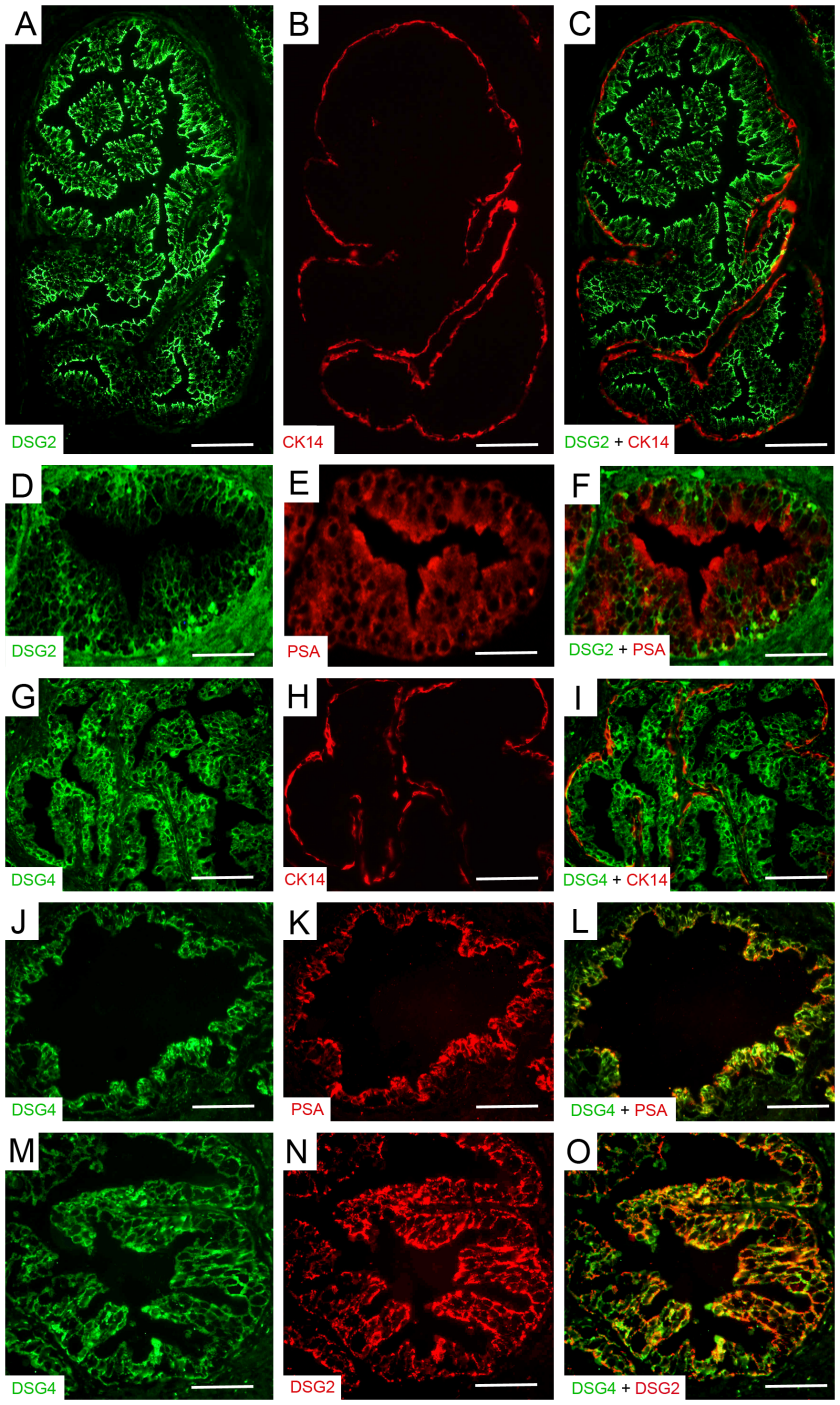
DSG2 and DSG4 are specifically localized in the luminal cells of the normal prostate gland. (A–C) DSG2 is expressed in the luminal cells of the prostatic glands and this expression rarely co-localizes with basal cell CK14 expression. (**D–F**) DSG2 co-localizes with PSA expression in the luminal compartment of the gland. (**G–I**) DSG4 is also expressed in the luminal cells and rarely co-localizes with basal cell CK14 expression. (**J–L**) DSG4 expression co-localizes with luminal cell PSA expression. (**M–O**) The expression of DSG4 also shows almost complete co-localization with that of DSG2 in the luminal cells. Original magnification: 200X. Scale bars correspond to 100 µm.

Co-immunofluorescence analysis was performed to examine the localization of DSG2 and DSG4 with CK14 specific basal cell marker as well as the luminal specific protein, PSA. Co-immunofluorescence analysis of DSG2 and CK14 shows that DSG2 is robustly expressed in the luminal cells of the prostate and that this expression rarely co-localizes with that of basal CK14 expression (Figure 2A–C). Analysis of DSG2 and PSA expression revealed co-localization of both biomarkers in the luminal cells (Figure 2D–F). The expression of DSG4 was very similar to that of DSG2, showing strong expression in the luminal cells, rarely co-localizing with basal CK14 expression (Figure 2G–I) but co-localizing with luminal PSA expression (Figure 2J–L). Furthermore, the expression of DSG2 showed almost complete co-localization with that of DSG4, with most luminal cells showing an even level of expression for both desmogleins and some cells showing a slightly higher expression level for one as compared to the other (Figure 2M–O). Taken together these results illustrate that the expression of DSG2 and DSG4 is mainly restricted to the luminal cells of the human prostate epithelial glands.

### DSG2 is Expressed in Human Prostate Cancer Cell Lines

**Figure 3:**
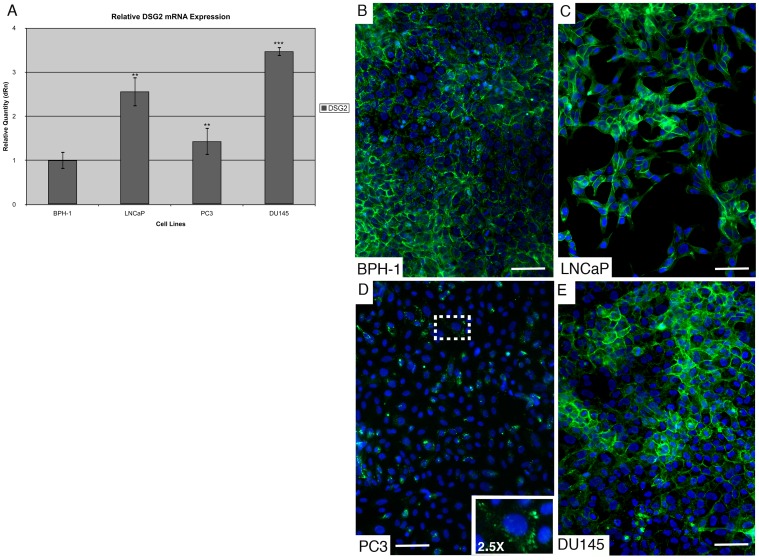
DSG2 expression in human metastatic prostate cancer cell lines. (A) DSG2 expression is detected in all cell lines examined, including the normal immortalized prostate cell line BPH-1. Data is represented as mean ± SD. **P<0.01; *** P<0.001. (**B–E**) DSG2 is expressed at the cell border of LNCaP and DU145 cells; however, cell border expression is not detected in PC3 cells with only some cells showing faint, punctate, and diffuse staining (insert magnification; shown at 2.5X). Original magnification: 200X. Scale bars correspond to 100 µm.

Having characterized the expression of desmogleins in normal human prostate tissue, we wished to focus on DSG2 for the remainder of the study as this is the most widely expressed desmoglein isoform — showing ubiquitous expression in all desmosome forming tissues. Following our previous discovery that DSG2 is expressed in normal human prostate epithelium, we then asked whether this protein is also expressed in human prostate cancer cell lines. To examine this question, qRT-PCR was performed on RNA extracted from three commercially available and well characterized metastatic human prostate cancer cell lines (Figure 3A). The cancer cell lines utilized for this portion of the study include the LNCaP cell line, which was derived from a prostate cancer that metastasized to the lymph node; the PC3 cell line, which was derived from a prostate cancer that metastasized to the bone; and the DU145 cell line, which was derived from a prostate cancer that metastasized to the brain [38]–[40]. The BPH-1 cell line served as a control for this study, since it is an immortalized, non-tumorigenic prostatic epithelial cell line derived from a patient with benign prostatic hyperplasia, a condition not related to malignant prostate transformation [41]. The results of this qRT-PCR analysis showed that *DSG2* is expressed at different levels in all cell lines examined. Interestingly, the expression of *DSG2* is greater in all the metastatic prostate cancer cell lines examined than it is in the non-tumorigenic BPH-1 control.

Next, immunofluorescence analysis was utilized to examine the expression of DSG2 in these metastatic human prostate cancer cell lines at the protein level (Figure 3B–E). Cell border expression of DSG2 was detected in BPH-1, LNCaP, and DU145 cells. However, PC3 cells lacked the expression of DSG2 at the cell border, while a small population of cells showed only a diffuse granular staining for DSG2 in the cytoplasm (see insert magnification in Figure 3D). These results are consistent with and further refine the results of a previous study by Lang *et al.* in which an antibody specific for both DSG1 and DSG2 was used to examine desmoglein expression in LNCaP, PC3, and DU145 cells [29], [42]. The results of the qRT-PCR and immunofluorescence analysis show that the expression of *DSG2* is present in all prostate cancer cell lines and that, in two of the three prostate cancer cell lines examined, DSG2 localizes to the cell border suggesting that desmosomal formation is retained in most metastatic prostate cancer cell lines examined *in vitro*.

### A Low DSG2 Phenotype is associated with Tumor Progression in Patients with Primary Prostatic Carcinoma

As mentioned previously, while the loss of classical cadherin expression has been described in prostate cancer, to date the expression of desmosomal cadherins in prostate carcinoma tissue samples has not been reported. To assess the percentage of cells positive for DSG2 expression in prostate carcinoma as compared to normal prostate tissue, immunofluorescence analysis of DSG2 expression was performed on prostate carcinoma TMAs (*n* = 414) as well as normal prostate TMAs (*n* = 50). In addition to an antibody for the protein of interest, an antibody for CK8/18 — cytokeratins found in both normal prostate luminal epithelial cells and adenocarcinoma cells — was also included on each TMA as a means of identifying the epithelial cells in each sample.

**Figure 4:**
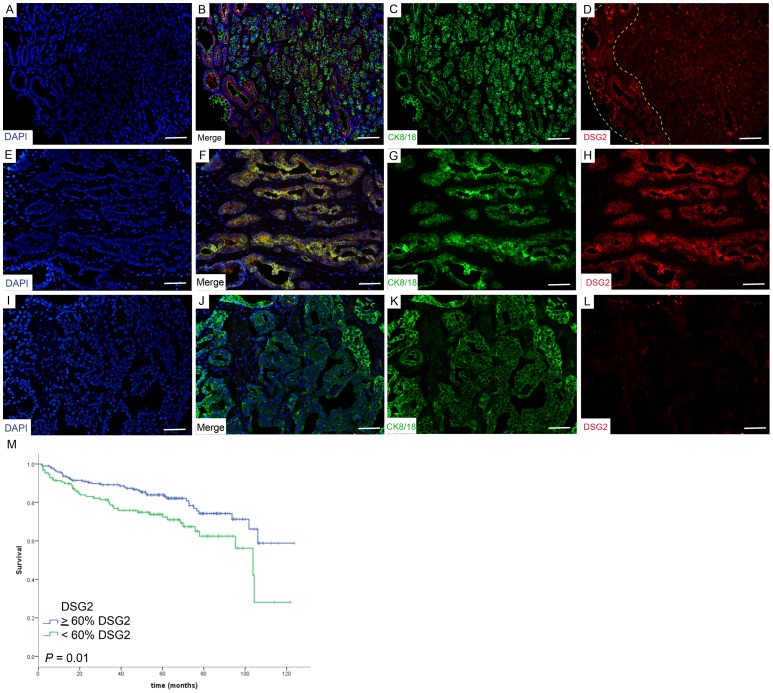
Low expression of DSG2 is a poor prognosis biomarker for patients with prostatic carcinoma. (A–L) Representative immunofluorescence analysis of DSG2 and CK8/18 in prostate cancer. (**D, E–H**) TMAs showing high DSG2 expression in the cell border of well differentiated areas of the tumor (panel D, inside yellow dashed line). (**D, I–L**) TMAs showing low DSG2 expression in poorly differentiated areas of the tumor (panel D, shown in the remainder of the tumor outside of the yellow dashed line). Original magnification: 200X. Scale bars correspond to 100 µm. (**M**) Patients expressing higher levels of DSG2 had a significantly longer recurrence free survival than those expressing lower levels of DSG2 (*P* = 0.01).

A significant decrease in the percentage of cells positive for DSG2 expression was found in prostate carcinomas as compared to normal prostate (Table 2 and Figure S3). Normal prostate epithelium was characterized by a high percentage of cells positive for DSG2 expression (mean expression of 81.1%, median of 84.5%), whereas prostate tumor samples showed a significantly lower percentage of DSG2 positive cells (mean expression of 57.5%, median of 66.7%; *P* = 0.0002). A cut-off value of 60% DSG2 positive cell expression was utilized, as this was the approximate median expression value observed for DSG2 in the prostate cancer cohort, and median has been previously and widely used to perform survival analyses [35]. Using this cut-off value, we observed that 96% of normal prostate tissue samples were considered positive for DSG2, in comparison to only 46% of the prostate carcinoma specimens studied. Notably, the amount of cells that were positive for cell border expression of DSG2 was generally higher in well differentiated areas of the tumor, while the amount of cells showing this expression was often much lower in poorly differentiated areas of the tumor (Figure 4A–L), a finding that was further validated by Spearman's rank correlation (Table 3).

**Table 2:** Expression of DSG2 in prostate cancer as compared to normal prostate.

DSG2 expression	Cancer	Normal	*P*-value*
**Mean**	57.5	81.1	0.0002
**Standard Deviation**	25.7	9.9	
**Median**	66.7	84.5	
**Interquartile Range**	40–76.7	80.0–86.7	

*P-value determined using Student's T-test.

**Table 3:** Correlation between DSG2 and clinico-pathological characteristics.

		PSA	Gleason Score	TNM Stage	DSG2
**PSA**	**Correlation**	1.000	0.294**	0.256**	−0.161**
	**Sig. (2-tailed)**	.	<0.0001	<0.0001	0.004
	**N**	410	400	410	316
**Gleason Score**	**Correlation**		1.000	0.363**	−0.122*
	**Sig. (2-tailed)**		.	<0.0001	0.031
	**N**		402	402	313
**TNM Stage**	**Correlation**			1.000	−0.084
	**Sig. (2-tailed)**			.	0.135
	**N**			412	318
**DSG2**	**Correlation**				1.000
	**Sig. (2-tailed)**				.
	**N**				320

*Spearman's rho correlation is significant at P<0.05 level and **P<0.01 level (2-tailed).

The correlation between the expression of DSG2 and the clinico-pathological characteristics most commonly associated with an aggressive prostate cancer phenotype, including serum Pre-surgical PSA concentration, Gleason Score, and Pathological Stage was then examined using Spearman's rank correlation (Table 3). Interestingly, there was a significant negative correlation between DSG2 expression and Pre-surgical PSA concentration as well as Gleason Score. These results revealed that a low-to-negative DSG2 expression phenotype was associated with increased serum Pre-surgical PSA concentration levels, as well as with high Gleason Scores.

### Decreased DSG2 Expression is an independent factor associated with a Poor Clinical Outcome in Patients with Primary Prostatic Carcinoma

Having examined the correlation between DSG2 phenotype and clinico-pathological risk factors of prostate carcinoma, we next determined whether DSG2 expression is associated with biochemical recurrence free survival. Interestingly, patients whose tumors expressed ≥ 60% DSG2 had a longer recurrence free survival than those expressing <60%, and the difference between these two groups was statistically significant (Figure 4M, *P* = 0.01). A median time to biochemical recurrence of 103.7 months (CI 95% interval: 87.7−119.8 months) was observed for patients with <60% DSG2 expression phenotype, while those with ≥60% DSG2 expression phenotype did not reach the median time to biochemical recurrence after a follow-up time of 123.6 months. More importantly, when we performed a multivariate analysis, we observed that DSG2 expression was an independent factor of biochemical recurrence (HR = 1.579, *P* = 0.050), as well as the classic clinico-pathological features: Gleason Score and Pathological Stage (Table 4). Taken together, these results show that reduced DSG2 expression is an independent biomarker associated with a shorter biochemical recurrence in prostate cancer, thereby revealing the potential clinical utility of DSG2 as a prognostic biomarker of tumor aggressiveness for patients affected with this frequently occurring disease.

**Table 4:** Multivariate analyses in the 414 patient cohort.

	HR*	95% CI**	*P*-value***
**DSG2**	**1.579**	0.996−2.503	0.050
**Pre-surgical PSA**	1.340	0.956−1.878	0.089
**Gleason Score**	**2.036**	1.518−2.732	<0.0001
**Pathological Stage**	**2.296**	1.499−3.517	<0.0001
**Angiolymphatic invasion**	0.623	0.248−1.570	0.316
**Perineural invasion**	0.973	0.515−1.838	0.933

*HR: Hazard Ratio;

**CI: Confidence Interval;

***P-value determined by Cox proportional hazards model.

## Discussion

The results presented in this study provide the first molecular characterization of desmoglein expression in normal human prostate, characterization of DSG2 in metastatic prostate cancer cell lines, and the first analysis of DSG2 expression in tissue samples of primary prostatic carcinoma. The results here reported reveal that while most desmogleins are distinctly expressed in the human prostate at the RNA level, only DSG2 and DSG4 are consistently identified at a high level in normal human prostate at the protein level. Further, the expression of DSG2 and DSG4 was found to be largely restricted to the luminal cells of the prostatic glands. Prior examination of DSG4 has shown that DSG4 is expressed in the most mechanically stressed layers of the epidermis and hair shaft, thereby suggesting that this desmoglein may provide stronger adhesion than the other desmoglein isoforms. The presence of DSG4 in the prostatic glands may similarly provide an additional level of adhesive strength in the desmosomes of luminal cells beyond that provided by DSG2 alone. While an examination of all desmogleins in prostate cancer was outside the scope of this study, future examination of the remaining desmoglein family members would likely yield a valuable understanding of the role of desmosomal adhesion in prostate cancer initiation and progression.

Interestingly, the expression of DSG2 is present in metastatic prostate cancer cell lines *in vitro*. This observed expression of DSG2 is in line with a previous study conducted by Trojan et al. in which microarray analysis revealed that *DSG2* is overexpressed in the highly metastatic LNCaP-C4-2 cell line as compared to the less metastatic LNCaP cell line [28]. Taken together these results may reflect the need for a re-expression of DSG2 in an aggressive cancer in order to establish the cell-cell adhesion necessary to form a metastatic tumor.

There is a lack of functional evidence regarding the role of desmosomal cadherin based adhesion in cancer. However, *in vitro* analysis of a non-adhesive, invasive fibroblast cell line showed that the expression of desmosomal cadherins and plakoglobin was able to generate adhesion and reduce the invasive capacity of the cell line [15]. Treatment of these cells with peptides that blocked the desmosomal cadherin adhesion site both blocked adhesion and restored invasive capacity, suggesting that the observed adhesion and reduced invasive capacity were desmosomal cadherin specific. These results suggest a tumor suppressor role for desmosomal cadherins and illustrate the possibility that desmosomal adhesion may result in a similar impaired invasive capacity of cancer cells, and that the loss of this adhesion may be required for the progression of the disease. However, this tumor suppressor role is controversial as *in vivo* analysis of transformed Dsg3^−/−^ keratinocytes showed impaired tumor growth, thereby suggesting an alternative tumor promoting role for desmosomal cadherins [43].

Prior to this study, the expression of desmogleins in prostate cancer tissue samples had not been reported. Given that desmosomes are involved in cell-cell adhesion in prostatic epithelium, an understanding of the expression of desmogleins would provide a deeper insight into the role of anchoring junctions in prostate cancer progression. Interestingly, DSG2 expression was significantly reduced in prostate cancer as opposed to normal prostate. Specifically, the expression of DSG2 was generally high in well differentiated areas of the tumor (low Gleason Score) and low in poorly differentiated areas of the tumor (high Gleason Score). Consistent with this observation, the results of the Spearman's rank correlation show that there is a negative correlation between DSG2 phenotype and serum Pre-surgical PSA concentration, Gleason Score, and Pathological Stage. Taken together these results suggest that DSG2 expression is reduced in primary prostate tumors with an aggressive biological behavior. Furthermore, the results of the Kaplan-Meier biochemical recurrence free survival curves denote that patients with a reduced DSG2 expression phenotype have a worse prognosis and poorer clinical outcome than patients whose prostate carcinoma is characterized by a high DSG2 expression phenotype; a finding further confirmed by multivariate analysis. In sum, these results highlight a potentially critical role for DSG2 based cell-cell adhesion in the progression of prostate carcinoma, and demonstrate that DSG2 may be a useful predictive biomarker of clinically significant prostate cancer.

## Supporting Information

Figure S1 **Biochemical recurrence free survival curves for the classical prostate cancer recurrence risk factors in the studied cohort of 414 patients.** (A) Pre-surgical PSA, (**B**) Pathological Stage, (**C**) Gleason Score, (**D**) Angiolymphatic Invasion, and (**E**) Perineural Invasion.
(TIF)

Figure S2 **DSG1 and DSG3 are not expressed in normal prostate.** (A–C) Representative DSG1 expression in a normal prostate gland. (**D–F**) Representative DSG3 in a normal prostate gland. DAPI is depicted in left panels, DSGs in center panels, merged images in right panels. Original magnification: 200X. Scale bars correspond to 100 µm.
(TIF)

Figure S3 **DSG2 differential expression in normal prostate and prostate cancer.** Box plot illustrates that DSG2 is significantly expressed at higher levels in normal prostate as compared to prostate cancer.
(TIF)

Table S1 **RT-PCR Primers and qRT-PCR Primers.**
(DOC)
