# Supplementary figures and images for: Characterization of Desmoglein Expression in the Normal Prostatic Gland. Desmoglein 2 Is an Independent Prognostic Factor for Aggressive Prostate Cancer
Source: PLoS One. 2014 Jun 4;9(6):e98786. doi: 10.1371/journal.pone.0098786 (PMC4045811; doi:10.1371/journal.pone.0098786)

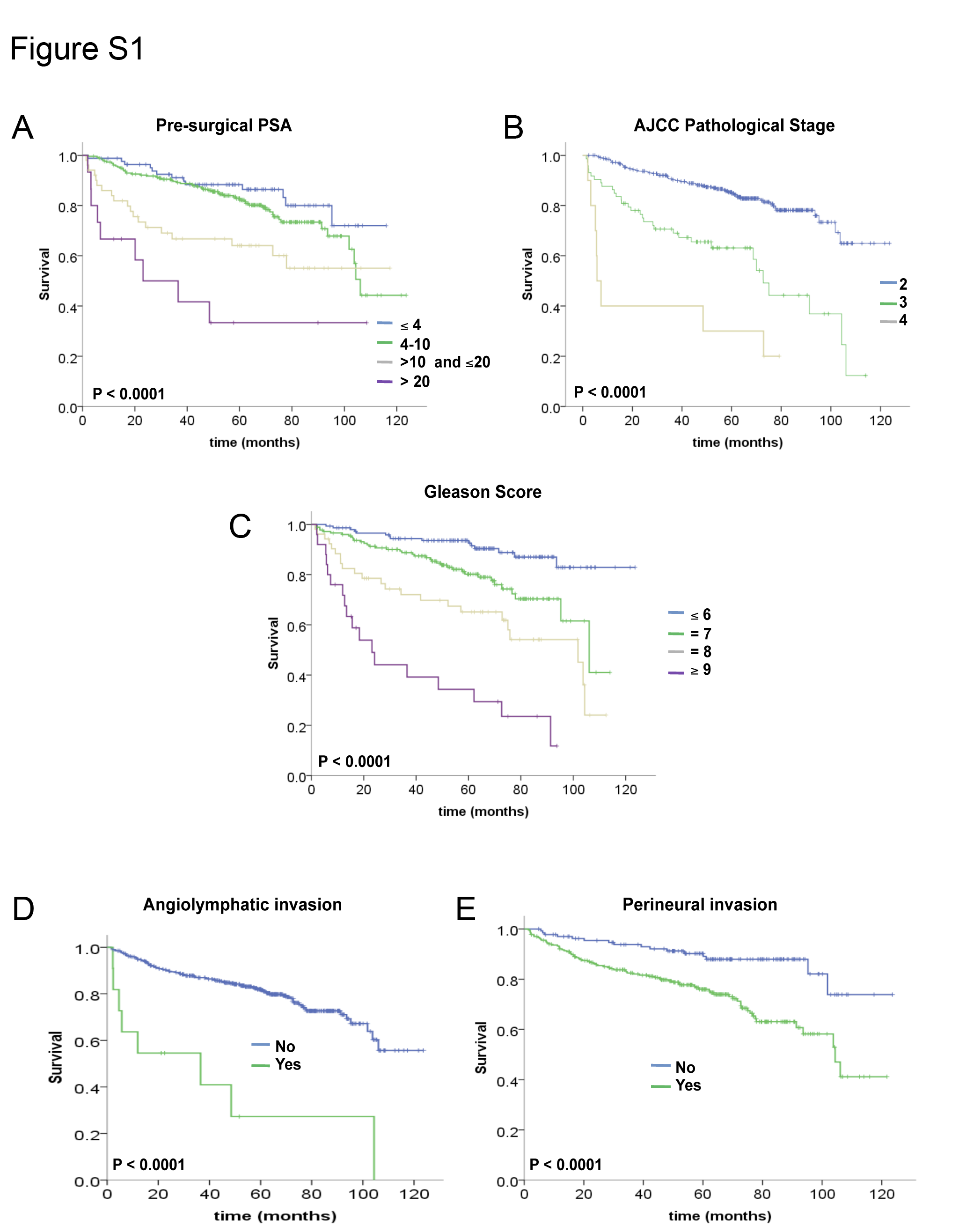

Supplement: Figure S1 — Biochemical recurrence free survival curves for the classical prostate cancer recurrence risk factors in the studied cohort of 414 patients. (A) Pre-surgical PSA, (B) Pathological Stage, (C) Gleason Score, (D) Angiolymphatic Invasion, and (E) Perineural Invasion. (TIF) [file pone.0098786.s001.tif]

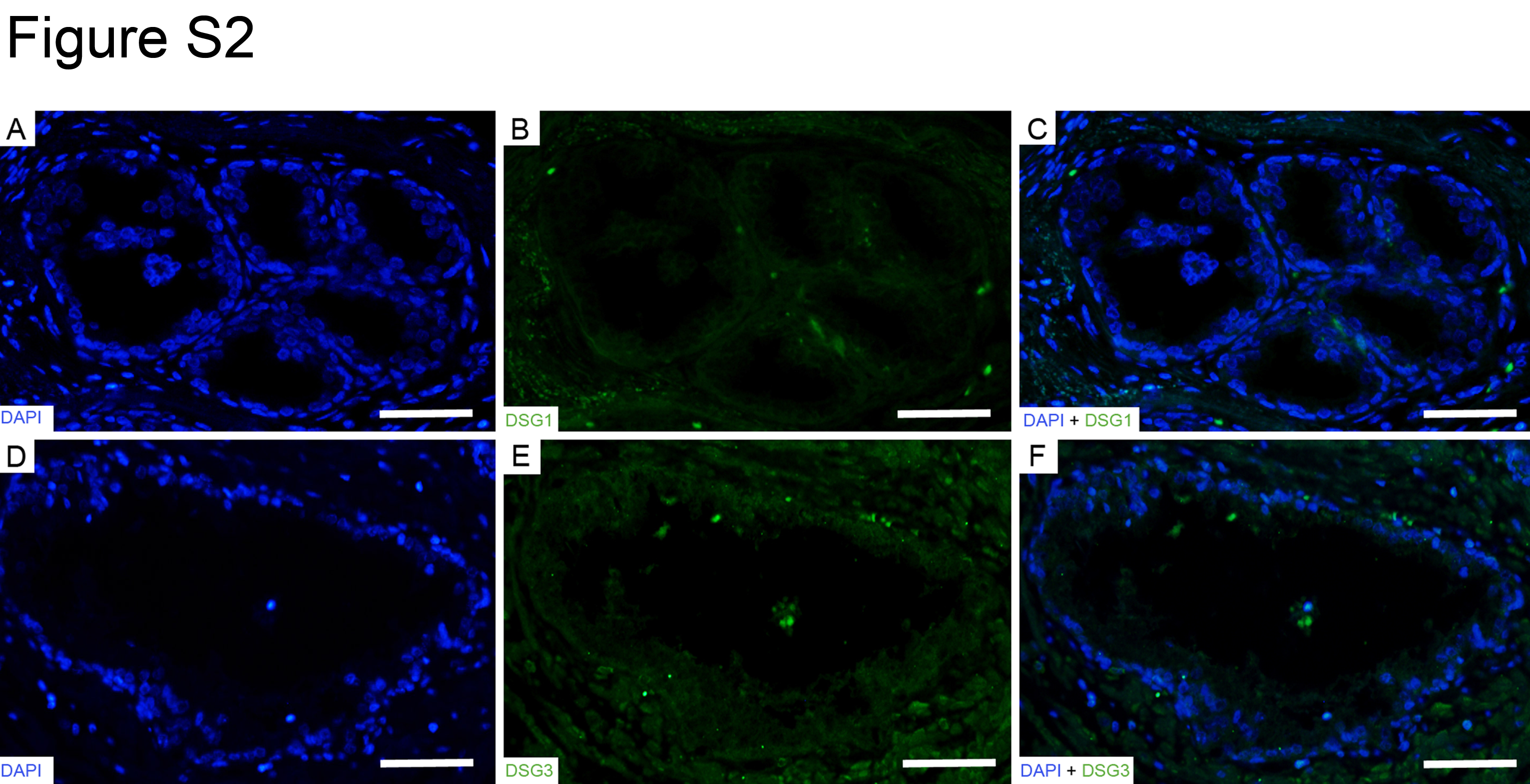

Supplement: Figure S2 — DSG1 and DSG3 are not expressed in normal prostate. (A–C) Representative DSG1 expression in a normal prostate gland. (D–F) Representative DSG3 in a normal prostate gland. DAPI is depicted in left panels, DSGs in center panels, merged images in right panels. Original magnification: 200X. Scale bars correspond to 100 µm. (TIF) [file pone.0098786.s002.tif]

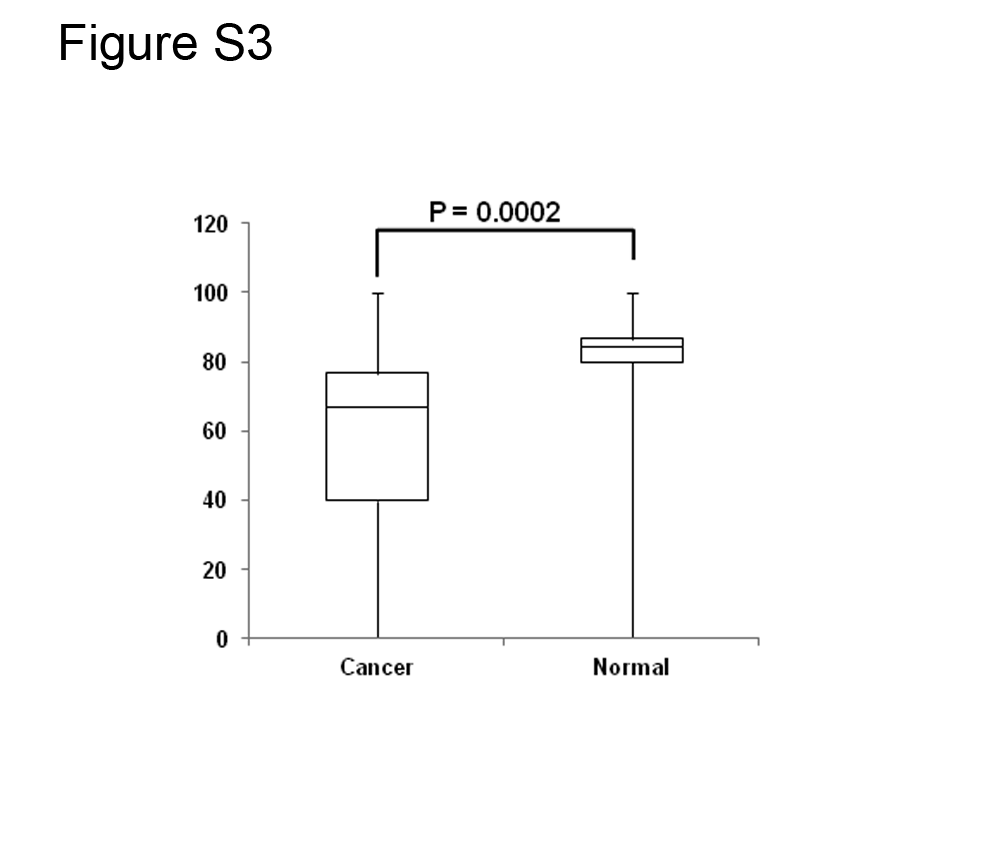

Supplement: Figure S3 — DSG2 differential expression in normal prostate and prostate cancer. Box plot illustrates that DSG2 is significantly expressed at higher levels in normal prostate as compared to prostate cancer. (TIF) [file pone.0098786.s003.tif]
