# Supplementary material for: Characterization of Desmoglein Expression in the Normal Prostatic Gland. Desmoglein 2 Is an Independent Prognostic Factor for Aggressive Prostate Cancer
Source: PLoS One. 2014 Jun 4;9(6):e98786. doi: 10.1371/journal.pone.0098786 (PMC4045811; doi:10.1371/journal.pone.0098786)
Supplement: Table S1 — RT-PCR Primers and qRT-PCR Primers. (DOC) [file pone.0098786.s004.doc]

**Table S**1: RT-PCR Primers and qRT-PCR Primers

|  | **RT-PCR Primers** |  |
| --- | --- | --- |
| Transcript | Forward Primer | Reverse Primer |
| DSG1 | 5’-CACTCAGATTGTGCTGCAAAC-3’ | 5’-GTCCTGCAAATGTAGCCATTG-3’ |
| DSG2 | 5’TCTTGAGGCCCTATGCAGTT-3’ | 5’-GCTGCACTCAACTCTTCAAC-3’ |
| DSG3 | 5’-GAGATGACTATGCAACAAGCT-3’ | 5’-TTCTCTACATCTAGTCCTTGG-3’ |
| DSG4 | 5’-ATGGATTGGCTCTTCTTCAGA-3’ | 5’-ACTCTAAGCTCAAGAGGCCT-3’ |
| β-actin | 5’-GATGATGATATCGCCGCGCT -3’ | 5’-CCTGGATAGCAACGTACATG-3’ |
|  | **qRT-PCR Primers** |  |
| DSG2 | 5’-ATCAATGCAACAGATGCAGATGA-3’ | 5’-TGTCAAAGTGTAGCTGCTGTGT-3’ |
| β-actin | 5’-AAACTGGAACGGTGAAGGTG-3’ | 5’-GTGGCTTTTAGGATGGCAAG-3’ |
